# Standardized Neonatal ICU Progress Note Template and Feedback System: A Clinical Documentation Improvement Initiative

**DOI:** 10.7759/cureus.75309

**Published:** 2024-12-08

**Authors:** Fahad Butt, Nisha Varghese, Ahmed Elhadidi, Sarah Abdulrahman, Aimen Ben Ayad

**Affiliations:** 1 Neonatology, Tawam Hospital, Al Ain, ARE; 2 Pediatrics, Tawam Hospital, Al Ain, ARE

**Keywords:** clinical documentation audit, clinical documentation improvement, errors in documentation, neonatal care, neonatal intensive care unit (nicu), nicu documentation, pediatrics and neonatology, plan-do-study-act (pdsa), quality improvement and patient safety, quality improvement projects

## Abstract

Introduction

This quality improvement (QI) initiative aimed to improve the clinical documentation of daily progress notes in the neonatal intensive care unit (NICU) by applying a standardized documentation template and conducting regular cycles of audit and feedback to ensure compliance and improvement.

Methods

Firstly, to better assess documentation practices impacting patient care, members of the NICU auditing team identified seven key points in medical records. These points were then used for the audit of 30 randomly selected "progress notes" for infants admitted to the NICU between January and June 2022.

We introduced a new standardized progress note template in the NICU based on the initial seven key points considered essential in NICU documentation. Subsequently, we educated the staff on the latest changes and their impact on patient care delivery. Also, we raised awareness among the NICU staff regarding the quality improvement project we were running and the requirement to adopt the new standard template. We also ensured that everyone had access to the new template.

The QI team analyzed NICU progress notes every three months from October 2022 to July 2023; 45 notes were reviewed per cycle, a total of 135 notes. With each cycle, we took feedback from NICU team members regarding the deficiencies and opportunities for improvement in clinical documentation and encouraged adherence to key points. A template for the specific needs of the NICU was redesigned based on feedback received from all stakeholders on how to reduce the obstacles in adopting and retaining compliance with the new template.

Results

Compliance with the "key elements" of documentation improved dramatically after introducing a new template and feedback system. In the final cycle, the overall compliance was 81% (p<0.0001), well above our initial target of 60% compliance.

Conclusion

A unit that implements targeted audit-and-feedback measures relevant to the clinical team requirement can substantially and consistently improve documentation.

## Introduction

A daily progress note is a document that illustrates and summarizes the main problems for which the patient is under care, as well as the treatments and investigations completed thus far, in a systematic and organized manner. It should be precise and easy to follow and include details necessary for an accurate management plan.

In a neonatal intensive care unit (NICU), it is essential to keep a daily progress note that clearly links all significant occurrences without creating confusion. Given the often extended and intricate nature of NICU admissions, the risk of documentation mistakes is significantly increased, which can result in misguided management strategies and pose risks to the infant's health.

The degree to which these documentation inconsistencies relate to adverse patient outcomes remains unclear [1]. Nevertheless, considering the critical role of precise information in making clinical choices for severely ill newborns, any inconsistencies or inaccuracies should be viewed as a possible source of error [2]. Having a standardized progress note can play a crucial role in mitigating these risks. A study by Cohen et al. found variation in the quality of documentation between healthcare providers [3]. This inconsistency might result in inadequate documentation and the possibility of patient harm from overlooked or incorrectly understood information. Consequently, minimizing this variability could also be seen as important [4].

Recognizing that incomplete documentation compromises patient safety, we initiated a quality improvement (QI) project. Overall, we aimed to create a standardized template for daily progress notes in the neonatal ICU by consensus with the help of a dedicated team who conducted regular audit cycles and took regular feedback to improve the template as per the unit's requirements and, therefore, improve clinical documentation.

## Materials and methods

Objective

Our objective was to increase overall average compliance to "key points" in clinical documentation, which would improve the quality of documentation in the electronic medical records (EMR), from a baseline of 35% to 60% within 12 months.

Methods

We conducted the study in the NICU at Tawam Hospital, Abu Dhabi, United Arab Emirates (UAE), which serves as the tertiary NICU for the Al Ain region, with more than 500 admissions per year. The multidisciplinary team consists of a neonatologist, neonatal hospitalist or fellow, pediatric resident, respiratory therapist, dietician, and pharmacist, with documentation managed electronically via the Cerner electronic medical record system.

The NICU auditing team identified key documentation points that directly impact patient care (see Table 1) and audited 30 randomly selected daily progress notes from January to June 2022. The audit revealed that the primary notes were deficient in all key areas, resulting in an overall baseline compliance rate of approximately 35%.

**Table 1:** Key points in clinical documentation

Number	Key points
1	Active problem-based system review
2	Notes with consistent information
3	Notes without redundancy
4	Notes with only approved abbreviations
5	Updated past events in system review
6	Updated physical examination
7	Updated daily management plan

Definitions

We defined the "key points," as outlined in Table 1, as follows: daily progress notes were expected to summarize all key points necessary for the patient care plan each day, in accordance with our institution's requirement for daily documentation on all patients. Additionally, we focused on whether these notes were organized by the patient's active problems or issues. This approach would help in efficiently identifying and addressing any unidentified or unaddressed issues in NICU patients.

We also established criteria for the quality of notes. A note was considered to have consistent information when there were no discrepancies present. Conversely, if a note contained duplicated information, it was marked as having redundancy. Furthermore, our institution has shared a list of approved abbreviations with all healthcare professionals, and any use of unapproved abbreviations was tallied and noted. Lastly, we evaluated the completeness of the daily progress notes by checking for appropriate and significant updates related to past events, physical examinations, and management plans. If any of these updates were lacking, the respective points were marked as incomplete.

Study of the interventions

Our QI team consisted of one consultant, one specialist (NICU hospitalist) or fellow, and one or two residents.

**Figure 1:**
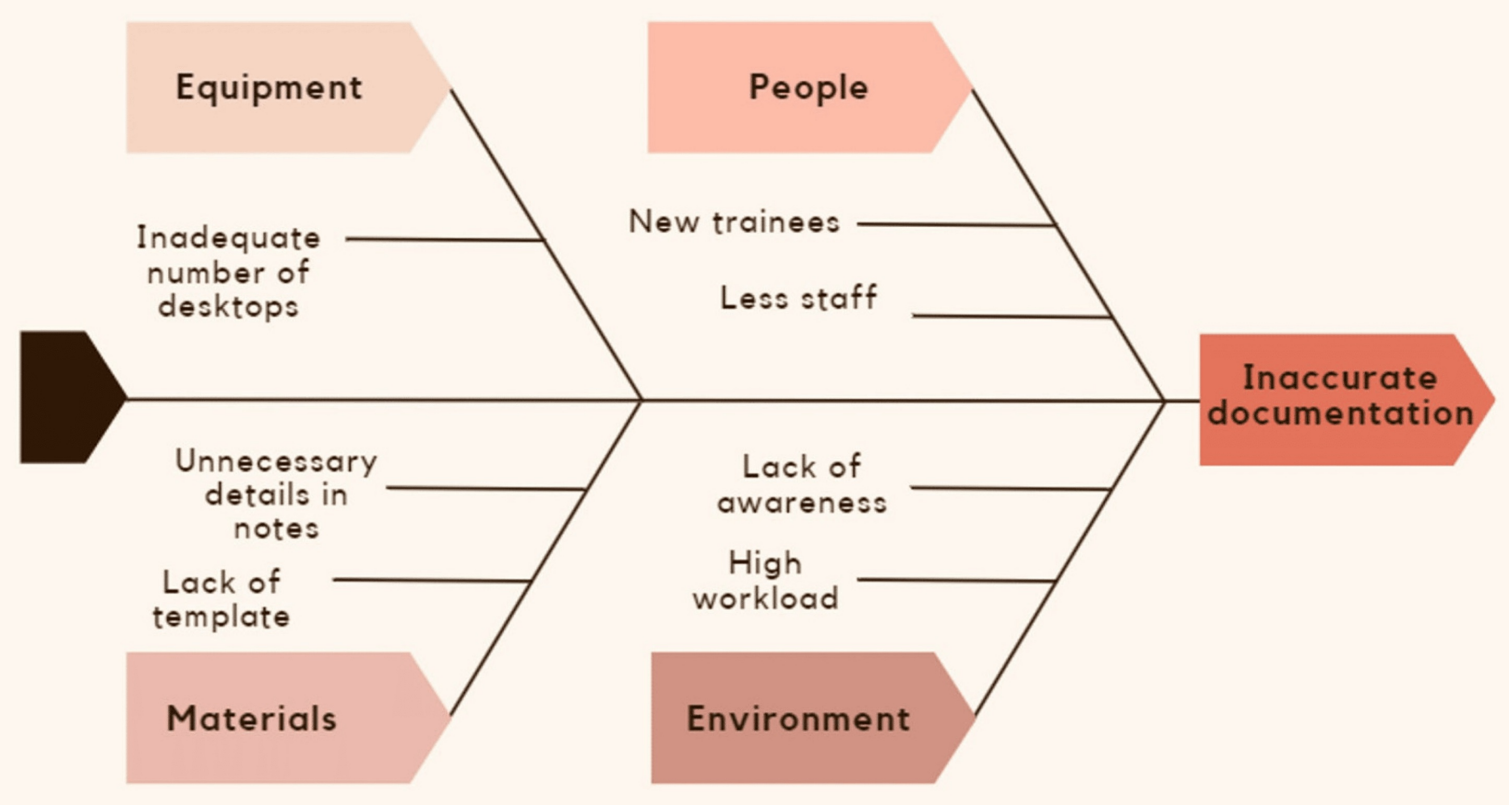
Ishikawa diagram of the project

All the project members agreed on definitions of "key points." Before the study, we identified potential barriers to achieving the goal as seen in Figure 1. We initially did a pilot audit and then regularly compared auditing techniques among us to keep the assessment consistent. There were four Plan-Do-Study-Act (PDSA) cycles as seen in Table 2.

**Table 2:** Plan-Do-Study-Act (PDSA) cycles NICU: neonatal intensive care unit

Cycles	Time	Plan	Do	Study	Act
PDSA 1	January to June 2022	Develop a standardized note	Dispersed instructions on how to use the new template	Poor compliance in using new template and the persistent use of unapproved abbreviations	Regular weekly email and in-person reminders
PDSA 2	October to December 2022	Notes redesigning to cater for the specific NICU team needs. List of approved abbreviations were redistributed	NICU physicians updated regarding the new changes in notes	Ongoing audit of charts	Weekly reminders and one-to-one feedback
PDSA 3	February to April 2023	Start regular orientation sessions for rotating residents	Actively sought feedback from all NICU physicians	Educated new physicians on the documentation of appropriate NICU events	Developed sustainable methods on educating physicians
PDSA 4	May to July 2023	Development of teaching manual to orient all new pediatric residents	Circulated the teaching document with all trainees via email	Evaluation of feedback and discussion of charts with NICU physicians	Regular audit for compliance

The first PDSA cycle involved an audit to identify necessary changes and develop a strategy. We created standardized daily progress notes to ensure uniform documentation, streamlining the auditing process. The project team met individually with NICU physicians to introduce the new template and communicated via email regarding its integration. In the second cycle, we addressed initial reluctance to adopt the new note due to ambiguities in the template's headings by simplifying the points and adding details, along with blank spaces for patient-related information.

The third PDSA cycle focused on the high turnover among pediatric residents in the NICU. The QI team conducted regular orientation sessions at the start of each rotation, improving residents' adaptation to the template and enhancing documentation consistency. In the fourth cycle, we created a teaching document to orient incoming residents and formally train them in proper documentation, addressing the high volume of daily progress notes and streamlining the orientation process.

In each cycle, we gathered feedback from NICU team members on clinical documentation improvements and encouraged adherence to key points.

Measures

The primary outcome was compliance with all key points, as depicted in Table 1.

Analysis

We collected data from January 2022 to July 2023 and recorded it in Excel sheets (Microsoft Corp., Redmond, WA). Data was audited and monitored at the end of each cycle by members of the QI team. We used the chi-square test to compare proportions, and a p-value of <0.05 was considered for statistical significance.

Ethical considerations

Tawam Hospital Review Committee reviewed this project and provided approval. We removed patient identifiers from all data collected and teaching cases.

## Results

We evaluated 165 notes, 30 pre-intervention and 135 post-interventions. The notes were randomly selected. The first cycle was an audit, which consisted of 30 notes. Then, each PDSA cycle lasted three months, with 45 notes in each cycle.

NICU physicians, fellows, and residents wrote notes. Compliance with our first key point reached 100% (n=45) by the second cycle and maintained 100% (n=45) until the last cycle. The second and third points, which audited consistency and redundancy in notes, respectively, also improved dramatically from 40% (n=12) and 30% (n=9) to 80% (n=36) and 84% (n=38) in the final cycle, respectively. Compliance with approved abbreviations, the fourth point, increased from 0% in the first cycle to 67% (n=30) in the second cycle, but in the latter two PDSA cycles, we saw a modest drop to 51% (n=23) and 58% (n=26), respectively. Up-to-date past events, physical examination, and management plan were the final three points. All three points noted improvement from a baseline of 36% (n=11), 66% (n=20), and 73% (n=22) to 87% (n=39), 70% (n=32), and 93% (n=42), respectively. The improvement of all points from baseline to the final cycle (36% to 81%) was statistically significant with a p-value of <0.0001 as seen in Table 3.

**Table 3:** Analysis of compliance to "key points" Values in the table are presented as n (%). P-value is considered significant if <0.05 *Significant p-values

		January to June 2022	October to December 2022	February to April 2023	May to July 2023	P-value
Number	Key points	Baseline (total notes: 30)	Cycle 1 (total notes: 45)	Cycle 2 (total notes: 45)	Cycle 3 (total notes: 45)	
1	Active problem-based system review	2 (6.7)	36 (80)	45 (100)	45 (100)	<0.0001*
2	Notes with consistent information	12 (40)	35 (78)	37 (82)	36 (80)	<0.0001*
3	Notes without redundancy	9 (30)	39 (87)	38 (73)	38 (84)	<0.0001*
4	Notes with only approved abbreviations	0 (0)	30 (67)	23 (51)	26 (58)	<0.0001*
5	Updated past events in the system review	11 (36)	40 (89)	35 (78)	39 (87)	<0.0001*
6	Updated physical examination	20 (66)	29 (64)	33 (73)	32 (70)	0.801
7	Updated daily management plan	22 (73)	41 (91)	42 (93)	42 (93)	0.02*
	Total	76 (36)	250 (79)	253 (78)	258 (81)	<0.0001*

## Discussion

Standardized progress note templates reduce inaccurate documentation. We demonstrated this by wholesome improvement in all key documentation points, which surpassed our initial target with statistical significance demonstrated in almost all domains. Overall compliance to seven key points in baseline data (January to June 2022), as seen in Table 3, was only 35%, and in the third and final cycles, the compliance was 81%, well above our initial target of 60% (p<0.0001). We showed evidence that a strategic audit of relevant key points in documentation, continuous bidirectional feedback, regularly addressing all stakeholders' concerns, and devising ways to address these issues could significantly improve clinical documentation quality, which in turn can positively affect the quality of patient care.

We found that a significant challenge in units with a high turnover of new residents and new trainees was the continuous education of new arrivals on appropriate documentation relevant to the unit. We addressed it sustainably by developing and circulating a teaching manual to orient all new staff regarding standardized templates and desired required points in documentation. This was also demonstrated in a different study where a systematic, periodic feedback from faculty to residents regarding patient documentation helped improve the quality of clinical notes [5].

The core purpose of electronic health-generated documentation should be to produce clear, history-driven entries that help shape an evaluation, along with a diagnostic and treatment strategy [6]. It was emphasized as early as 1968 when Dr. Weed highlighted the significance of the problem-oriented note and the organization of clinical information [7]. More recently, a study by Liu and Walsh in 2018 demonstrated that problem-based NICU documentation can positively improve documentation [8]. This should be done without creating lengthy notes. In their paper, Schiff and Bates pointed out that the issue of excessive information is now exceeding the problem of having insufficient data [9].

Regular audits of notes are crucial for maintaining their quality. Each unit should tailor its approach to evaluating note quality to best fit its specific needs. The quality of physician notes has been characterized in different ways, such as by the presence of key elements such as completeness or accuracy [10], as well as through subjective evaluations of their format and substance [11]. A study conducted by Tang et al. focused on evaluating note quality by assessing the inclusion of problem lists, medication lists, and a relevant assessment and plan [12].

Our QI study aimed to enhance the quality of daily progress notes using a standardized template. This approach was also seen in a QI project by Goswami et al., which aimed at improving cerebral function monitoring (CFM) documentation. One of the strategies they implemented, similar to ours, was the standardization of the CFM note. Their results demonstrated a statistically significant improvement in all aspects of documentation quality [13]. The use of a template was emphasized in a study by Grogan et al., which found that using a progress note template improves the documentation of key issues and complications [14]. In 2022, Durrani et al. also highlighted that accurate and thorough daily progress note documentation can lead to a more precise discharge note, ultimately allowing for better time allocation for other tasks on the unit [15].

Strengths

The strengths of our study lie in the successful implementation of a robust quality improvement strategy, which led to significant improvements in compliance with key documentation elements. Introducing a comprehensive teaching manual and regular orientation sessions proved highly effective in addressing the challenges of high staff turnover. Moreover, the structured approach employed, utilizing Plan-Do-Study-Act (PDSA) cycles, offers a strong and replicable model for quality improvement.

Limitations

The study was conducted at a single center in the UAE with unique resources, which may require adaptation when applied to other settings. We acknowledge the potential for subjectivity in the feedback received from stakeholders. While compliance improved during the study period, long-term adherence and sustained improvements were not evaluated.

## Conclusions

We provided evidence that significant improvement in clinical documentation can be achieved by developing auditing measures relevant to the clinical team and by building a documentation improvement team that can thoroughly audit and work with all partners regularly to achieve the unit's documentation improvement objective. We also highlighted that familiarizing new trainees with the standardized progress note template is imperative to attain the unit's QI documentation objective.
